# “It’s another gay disease”: an intersectional qualitative approach contextualizing the lived experiences of young gay, bisexual, and other sexual minoritized men in the United States during the mpox outbreak

**DOI:** 10.1186/s12889-024-19062-z

**Published:** 2024-06-11

**Authors:** Bryce Puesta Takenaka, Sally J. Kirklewski, Frances J. Griffith, Jeremy J. Gibbs, Carolyn K. Lauckner, Erin Nicholson, Cecil Tengatenga, Nathan B. Hansen, Trace Kershaw

**Affiliations:** 1 Yale School of Public Health, Department of Social and Behavioral Sciences, 60 College Street, New Haven, CT 06510 USA; 2 Yale School of Medicine, Division of Prevention and Community Research, 389 Whitney Avenue, New Haven, CT 06516 USA; 3 School of Social Work, University of Georgia, 279 Williams St, Athens, GA 30602 USA; 4 College of Medicine, Department of Behavioral Sciences, University of Kentucky, 800 Rose Street MN 150, Lexington, KY 40506 USA; 5 School of Medicine, University of Connecticut, 200 Academic Wy, Farmington, CT 06032 USA; 6 Department of Health Promotion & Behavior, University of Georgia College of Public Health, 100 Foster Road, Athens, GA 30606 USA

**Keywords:** Gay, Bisexual, Sexual minoritized men, Mpox, Stigma, Intersectionality

## Abstract

**Background:**

The U.S. mpox outbreak in 2022 introduced new and exacerbated existing challenges that disproportionately stigmatize gay, bisexual, and other sexual minoritized men (GBSMM). This study contextualizes the perceptions, susceptibility, and lived experiences of the mpox outbreak among GBSMM in the U.S. using an intersectional framework.

**Methods:**

Between September 2022 to February 2023, we conducted 33 semi-structured qualitative interviews with purposively sampled GBSMM in the Northeast and the South region of the United States on various aspects related to their experience during the mpox outbreak.

**Results:**

We identified four themes: (1) understanding and conceptualizations of mpox, (2) mpox vaccine availability and accessibility, (3) mpox vaccine hesitancy and mistrust, and (4) call to action and recommendations. GBSMM collectively discussed the elevated mpox stigmatization and homophobic discourse from mainstream social media and news outlets. GBSMM also discussed the lack of availability of mpox vaccines, unclear procedures to receive the vaccine, and continued mistrust in government, non-government, and other institutions of health that were complicit in anti-LGBTQ + narratives related to mpox. However, they expressed that these challenges may be addressed through more LGTBQ + representation and leveraging ways to empower these communities.

**Conclusion:**

GBSMM have mpox experiences that are distinct and multifaceted. Effectively addressing mpox and mitigating public health emergencies for GBSMM requires prioritizing destigmatizing communication channels and vaccine distribution strategies by centering their stories and lived experiences to advance health equity.

**Supplementary Information:**

The online version contains supplementary material available at 10.1186/s12889-024-19062-z.

## Background

We are approaching two years since the onset of the mpox [1], where the U.S. has seen roughly 31,010 reported cases of mpox [2]. Although mpox is no longer considered a public health emergency, its afterlife participates in contemporary health inequities. The majority of mpox cases were amongst individuals who identified as gay, bisexual, and other sexual minoritized men (GBSMM) [2]. Distinctly, 95% of these cases were made up of cisgender men and 93% of those between the ages 21–55 years-old. From these cases, 38 mpox-associated deaths have been reported and about 95% of these deaths occurred among cisgender men. More than 592,000 men have also been predicted to live in jurisdiction with risk for mpox recurrence [3], yet recent findings indicate only 23% of men who have sex with men have been fully vaccinated [4]. Despite the mpox vaccines being free of charge, inequities in uptake persist. The infancy in mpox-vaccine literature have suggested that GBSMM experience fear and stigma-related challenges that facilitate hesitancy for seeking out the mpox vaccine [5–8].

Given the historically low occurrence of mpox in the U.S., urgent efforts to reduce the spread of mpox led to the implementation of large-scale health risk messaging. The predominant, but not exclusive, transmission of mpox among GBSMM propelled the development of preventative messaging tailored to this population. Through a convenience sample from the American Men’s Internet Survey, Delaney and colleagues (2022) reported roughly 48% of GBSMM reducing their number of sexual partners, 50% reducing one-time sexual encounters, and 50% reducing meeting partners on dating apps or sex venues since learning about the mpox outbreak [9]. These findings coincide with epidemiological trends of mpox where cases declined, emphasizing the importance of mpox messaging for communities to engage in public health preventative behaviors [10]. The dissemination of rapid mpox messaging may be partially responsible for the decrease in mpox cases, but consequentially resulted in anti-LGBTQ + discourse apparent through mainstream social media and news channels [11, 12, 13].

Coupled with the increased usage of mainstream news and social media, and persistent anti-LGBTQ + systems, mpox became the vector for disseminating online homophobic, offensive rhetoric, and conspiracy theories [14–17]. For instance, Hong and colleagues (2022) conducted ecological analyses of LGBTQ + stigmatizing Reddit messaging related to mpox, resulting in reports of blaming related to the spread of mpox, increased interpersonal stigma and negative mental health outcomes [18]. Similarly, Twitter (now called X) text-mining methods illustrated ecological trends of homophobic, cyberaggression, and mpox-related hate against GBSMM [19]. Recent qualitative studies have also reported consistent themes [6, 19–21]. For instance, Smith and colleagues (2024) reported that their sample of men from Australia experienced judgement and mpox-related stigma from healthcare providers [20]. Similarly, Witzel and colleagues (2024) reported that among their sample of men based in England, they discussed the stigmatization of mpox was reinforced through media and national health agencies [22].

Despite the growing body qualitative approaches examining the impact of mpox among GBSMM, a majority of these studies are restricted to large ecological analyses that are likely to omit the rich nuances of the sociopolitical context of mpox. Understanding the sociopolitical and intersectional context of mpox has critical implications related to the acceptability of public health mitigation measures, as well as accessing testing and treatment [21]. The experiences of GBSMM residing in the US are particularly important given the unique sociopolitical context of mpox in which stigma, homophobia, and health care are differently situated compared to the geographies most mpox studies have taken place. While various studies have suggested the pervasive stigmatization of mpox speak to larger systemic paradigms needing to be explored [5, 6, 20, 22, 23], little is still known about the ways in which systemic manifestations of discrimination have shaped the embodied health experiences of GBSMM and its impact on utilizing mpox related services.

### Intersectionality as an analytical tool to examine sociostructural factors on mpox stigma

In the U.S., gay, bisexual, and other sexual minoritized men are social groups within the broader LGBTQ + community, who continue to experience multiple pathways of systemic (e.g., heterosexism, homophobia, transphobia, genderism) and structural (e.g., anti-LGBTQ + laws, restricted working conditions, housing discriminations, hate crimes) oppression [24–26]. While no collective definition of mpox stigma exists, it can be conceptualized as the societal disapproval of mpox and a social group that manifests from larger sociostructural practices of labeling, stereotyping, and discrimination [27], which holds consistent implications for minority stress. Minority stress theory suggests that individuals of marginalized identities experience an accumulation of stressors of stigma and discrimination that result in adverse health outcomes [28]. However, this theory only poses a limited framing that obscures the interlocking pathways GBSMM might have experienced during the mpox epidemic.

While the official term of ‘intersectionality’ was coined by Black feminist legal scholar Kimberlé Crenshaw [29], its critical framework and analytical praxis has long-existed and evolved through the scholarship of Black feminist writers, activists, and social justice advocates [29–32]. Intersectionality at its core challenges us to interrogate the hegemonic power structures baked into society and critique existing authoritative assumptions of seeing (and moving through) the world. Intersectionality centers the examination of micro-level social identities and positions that are shaped and reinforced by interlocking macro-level sociostructural systems of power, privilege, and oppression (e.g., homophobia, transphobia, racism) that permeate throughout all aspects of life [31, 33]. Within the context of this study, GBSMM are arranged where gender, sexual orientation, socioeconomic status, and geography are reflected by larger systems of cis-heterosexism, homophobia, classism, and spatial marginalization. The application of intersectional praxis for understanding mpox stigma provides an opportunity to critically interrogate the dynamics of how underlying power and privilege intersects with GBSMM’s social positions on their experience during the mpox epidemic.

Using an intersectional stigma lens, coined by Black feminist scholar Michele Tracey Berger, is defined as the “total synchronistic influence of various forms of oppression which combine and overlap to form a distinct positionality” [34]. Here, intersectional mpox stigma poses that GBSMM are not only marginalized, and socially situated (nexus of gender, sexual orientation, class, geography), but also historically (e.g., HIV/AIDS) carry negative mpox-stigmatizing narratives that become embodied (e.g., drug use, within community serosorting, labeled as “dirty”/“immoral”) [35, 36]. GBSMM are positioned at the nexus of heterosexism and homophobia but also within particular gendered norms, sexualized stereotypes, microaggressions that can impair sexual health decision-making [28]. Due to these anti-LGBTQ + structures, GBSMM have also been socially labeled as sexually promiscuous and continue to have harmful encounters with healthcare providers [37, 38] and within their routine social spaces. GBSMM across urban/rural landscapes also comprise of heterogeneous social and material arrangements of experiences [37, 39]. The spatialization of power structures of sexual minority stigma have greatly informed health outcomes among GBSMM. For instance, studies have found that lesbian, gay, and bisexual people reported better mental health outcomes and reduced mortality in states with supportive LGTBQ + laws and policies along with lower levels of anti-LGBTQ + discrimination [40, 41], whereas higher levels of anti-LGBTQ + discrimination have been associated with risky sexual behaviors and increased barriers to healthcare [37, 42].

We suggest these formations of compounding power asymmetries have shaped and amplified negative intersectional mpox-stigma among GBSMM, while reinforcing deeply entrenched stereotypes and avenues for structural discrimination to continue to marginalize GBSMM. Therefore, the purpose of this study was to contextualize the lived intersectional experiences of mpox stigma among gay, bisexual, and other sexual minoritized men reside in small cities and towns during the mpox outbreak in the US.

## Methods

### Participants

This analysis included participants of *#MVMNT (Men’s Voices on Mapping, Neighborhoods, and Technology)*, a prospective cohort study of 382 HIV-negative young sexual minority men in small non-metropolitan areas in the Northeast and Southeast between 2018 and 2022. Eligible participants were man-identifying (including transgender men), between the ages 18 to 34 years old, self-reported negative HIV serostatus, English or Spanish speaking, and self-reported any of the following characteristics in the past 12 months: had unprotected anal sex (sex without a condom or sex not on PrEP); had sex under the influence of alcohol or drugs; had sex with a partner who is living with HIV, injects drugs, or has sex with multiple partners; had a sexually transmitted infection; or had a partner hit, kick, punch, slap or force them to have sex when they did not want to. A subset of participants (*n* = 33) was offered a chance to participate in a qualitative sub study on monkeypox and were included in the analysis. Recruitment involved using both existing participants who were still part of the larger study and those who have already completed it and declared an interest to be followed-up for future opportunities to participate in research. The research protocol and informed consent were approved by Yale University and the University of Georgia Institutional Review Board (IRB) (IRB# 2,000,021,580) prior to participation in this study.

### Procedures

Semi-structured interviews took place between September 2022 and February 2023. The recruitment for these interviews was initiated as a part of the larger *#MVMNT* study. The research team developed the interview guide and internally reviewed it together (see Supplementary Material for Interview Guide). The guide underwent iterative feedback by the #MVMNT study principal investigators, research coordinators, doctoral students, and research assistants. These questions sought to explore the following: (1) general perceptions of the mpox outbreak, (2) information sources, (3) personal susceptibility, (4) opinions on the mpox vaccines, and (5) overall perceptions on the impact of mpox on the LGBTQ + community. Interviews were allotted an hour and comprised of one facilitator. Interviews were remotely conducted via Zoom, audio-recorded, and transcribed verbatim. Transcripts were listened to and verified by the research coordinators and research assistants. Participants received $25 for their time and investment for these interviews.

### Data analysis

The semi-structured interview data were thematically analyzed using NVivo version 12 [43–45]. The research team participated in an initial open coding that involved a round of reviewing a sub-sample of the transcripts to develop a draft of the codebook. The first and second author, and six coders met bi-weekly to discuss the findings from each round of coding. Following independent coding sessions, one of the principal investigators, a research coordinator, doctoral student, and the coders met bi-weekly to engage in reflexive conversations on the assumptions and interpretations of the codes. Themes, subthemes, and definitions for codes were iteratively proposed and decided through both inductive and deductive reasoning. The first and second author co-collaborated on analyzing overlapping sections of the transcripts and proposed themes, then compared and reformed with the senior author. In addition to thematically analyzing the coded transcripts, we deployed intersectionality as an analytical lens to consider to how we were perceiving and drawing conclusions of social categories (e.g., gender, sexual orientation) and social relations (e.g., stigma, homophobia). Discussions on intersectionality are not often explicit so recognizing that these identities cannot be detached from the simultaneity of oppression and privileged, the transcripts (which was led by the first author) were analyzed in relation to power structures and the multiplicative construction of these experiences [33, 46, 47]. Context also mattered for this analysis, where our participants being sexual and gender minoritized groups suggests that anti-LGBTQ + structures of discrimination may articulate clearer interactions of oppressions than other societal relations. Thus, our analysis was attentive to such structures of power but also remained cognizant of capturing intergroup relations between and within these groups. The first author reviewed again all of the study’s transcripts to ensure he had coded all of the relevant texts into any codes that were created after the initial coding phase. Throughout this iterative analytical and interpretative phase of the study, we held bi-weekly meetings with the larger research team to discuss and refine the findings together.

### Researcher’s reflexivity

Throughout the development of the research protocol, interview guide, codebook, coding of the interviews, analyses and interpretation of the findings, the research team engaged in iterative and reflexive positioning. Under the supervision of two co-principal investigators, both who are White cisgender men, three research coordinators – one who identifies as an African immigrant and trans-non-binary and two White cisgender women, and one doctoral student led the development of the research protocol and conducted interviews. One research coordinator, four undergraduate students, one doctoral student co-developed the codebook and engaged in iterative coding of the transcripts. The first author, who is a Brown cisgender queer man and doctoral student from the U.S. worked through the analysis and interpretations iteratively with the senior author. His decisions regarding the coding and analysis were informed by his own relational experiences and perceptions formed prior and during the mpox outbreak as a queer man and academic researcher. All stages of the study involved multiple iterative conversations involving the larger research team. While some of us share similar identities and lived experiences with the participants in this study, we also come from privileged social locations as academic researchers that may influence the way we share and represent our participant’s stories. We move through this study as humble and non-experts of the routine issues and embodied experiences of the GBSMM within our study and the larger LGBTQ + community.

## Results

A total of 33 young (aged 18–34) GBSMM participated in interviews. Participants had an average age of 26 (SD = 4.2). The majority identified as gay (74%), following 19% as bisexual. About racialized as non-Hispanic white (70%) and graduated from college (52%). Half (56%) of the participants had an annual income of $50,000 or more and almost everyone had some form of health insurance (93.3%) (Table 1). Young GBSMM engaged in discussions regarding the mpox outbreak, mpox vaccine, and the role of various institutions that shaped their perceptions and experiences.

**Table 1:** Demographic Characteristics (*n* = 33)

Variable	*N* (%)
Age (mean, SD)	226.4 (4.2)
Gender	
Cisgender man Transgender	32 (96.9) 1 (3.1)
Sexual Orientation (missing = 2)	
Gay Bisexual Queer or other	23 (74.2) 6 (19.4) 2 (6.5)
Ethnoracial Identity	
Non-Hispanic White Non-Hispanic Black Hispanic/Latinx	23 (70.0) 4 (12.0) 6 (18.0)
Education (missing = 6)	
Some college Graduated from college Completed graduate school	6 (22.0) 14 (52.0) 7 (26.0)
Income (missing = 6)	
$19,999 or less $20,000 - $49,999 $50,000 or more	2 (7.4) 10 (37.0) 15 (55.6)
Health Insurance (missing = 3)	
No Yes	2 (6.7) 28 (93.3)

SD – standard deviation

### Intersectional mpox-related stigma on understanding and conceptualizing mpox

Participants’ understanding of mpox was often accompanied by the way the LGBTQ + community, particularly sexual minoritized men were at risk for mpox. Most participants described the centrality of the queer community and their perceived association with mpox acquisition.

*I know it’s a viral infection that can manifest in blisters along like various parts of the body, like across your skin. So you can have it on your face. But some people have it in other areas. And I know that there was a stigma…like there’s like kind of like this stigma being formed as well as like backlash against the stigma that it is something that predominantly affects…or like that it’s an STI which according to…it’s like the, I guess, backlash…it’s not an STI and it’s associated with like just the queer community in general.***– Black cisgender gay man**.

Men also described their experience with understanding mpox, in large part, due to the structural narratives using homophobic language by social media and news outlets. Particularly, men in our study expressed how mpox became another reason for further stigmatizing and reinforcing homophobia against the LGBTQ + community.

*I mean, for the most part, it kind of portrayed it as a gay disease, which wasn’t it was like the greatest thing to see. In general, you never want a disease like from any perspective or healthcare perspective or epidemiology or anything like that. You never want something to be portrayed on one demographic because its kind of outcasts that demographic.***– White transman**.

Men discussed the conceptualization of mpox clearly equating sexual identity with biological (mpox) risk, particularly how mpox was often associated with being a “gay disease” that only gay men who have sex with men could acquire. Not only did the men in our study discuss stereotyping of GBSMM as groups who inherently spread disease, their comments also speaks to larger historical and deeply systemic rationales that perpetuate the fictitious hierarchies that GBSMM are fundamentally inferior and a risk to the rest of society [48]. Mpox was also described in relation with the early phases of the HIV/AIDS epidemic, where both mpox and HIV continue to be neglected by the government and other institutions of public health and medicine.

*But I think it’s one of those like, “oh, only one person died and it’s only affecting the gays. Oh, it’s fine.” Like, there’s a reason that happens. And it’s the same political stuff that we saw in 2015 in Indiana with HIV and we saw in the eighties and with HIV.***– White cisgender gay man**.

Moreover, the sexualization of mpox stigma formulated and perpetuated by these institutions have enabled the proliferation of heterosexist conspiracies. The concept of “straight” privilege participants describe suggests how men who identified as ‘straight’ were at lower risk for acquiring mpox, while also reinforcing hyper surveillance of men part the LGTBQ + community.

*I just know that initially, you know, all the people on…most…a lot of people on Facebook, especially places like that, you could see some really nasty stuff happening with them, talking about gay people and a lot of misinformation and people that were ignorant in general like they just don’t understand how it works. I’ve seen literally people say in comments like, “Oh, I can’t get it, I’m straight.” Stuff like that. And you know, well, it’s almost like Smallpox so you can definitely get it. I mean, kids started to get it and stuff. And, you know, I mean, just like with HIV stuff, I mean, just as many straight people have it. If anything, probably more straight people have it. I don’t…I don’t know…I don’t…I don’t know the stats, but I mean, you know, if…if this was not…if this didn’t start with gay men and it started with like some straight people or something, then it would have been a totally different…it would have been a public issue.***–White cisgender bisexual man**.

Coupled with COVID-19, men note the difference in empathy people would receive in comparison with mpox. Particularly, men discussed these parallels in relation to anti-Asian violence and hatred for COVID-19 as comparable to anti-LGBTQ + discrimination with mpox. Here, participants explain the ways diseases become politicized and socially constructed through historical and contemporary discriminatory practices to exclude disliked subgroups of the population.

*It’s very similar to how folks who got COVID early on, I think were treated. “Get the hell away from me.” “What’s wrong with you?” Type thing. Rather than, “How can I help you?” Also, I think it’s one of those things where it’s visually kind of jarring to to see. And so that whereas with COVID, I think people were more willing to be supportive and loving and kind and there for people because you couldn’t see it by definition, here you can. And I think that that doesn’t help when it comes to who’s, who, who’s willing to give care. And I also think one thing from the stigma like, I think that plays a huge role in there, too, in terms of. People are willing to to look out for the folks who are sick*. **– White cisgender gay man**.

*Like, I mean even with COVID, not to say that this isn’t didn’t do that, but you saw a lot of like Asian side hatred towards Asian. And I think that. I don’t think it necessarily happened towards gay men as much, but it did portray it very much as a gay men’s disease. And it was. It was interesting to see.***– White Transman**.

The geographic location of participants situated their understanding and perceived susceptibility of mpox. Several men understood the burden and risk of mpox being most notable within large metropolitan cities.

*That it was predominantly in New York and affecting gay men and that it involves the blistering that was really painful, like all over the body.***– White cisgender gay man**.

In general, men described news outlets as agents for the potential reinforcing mpox-related stigma through misrepresented narratives and strategically blame gay men for the rise of mpox cases. As such, men felt that these sources were conflating mpox transmission and risk between gay men and adolescents; one participant expressed concerns for the potential reinforcing existing homophobic stereotypes of gay men. In fact, these fables that stereotype queer people as predators and untrustworthy deviants have long existed, and have been manipulated commercial entities like the media [49–51]. Thus, perpetuates not only historical manifestations of anti-LGBTQ + tropes but reinforces the formula of promiscuity, GBSMM, and mpox.

*So like what few things that I heard about it on NPR that I listen to practically every day whenever I get in the car. Like the language that they would use changed slightly, but it was still in that same era of like discriminatory language that made it seem like this one group of people were causing it and not more so that cause and effect type situation.***– Black cisgender queer man**.

*For a little while it seemed like it was focused on children, which was concerning to me because there’s always this perception among some people that like gay men, are perpetrators of child abuse, etc. And so I think that that kind of played into that stereotype.***– White cisgender gay man**.

### Intersectional mpox-related stigma on vaccine availability and accessibility

Barriers to vaccine availability and accessibility were reoccurring across most participants. One such barrier was the physical inaccessibility of mpox vaccines due to only certain venues (e.g., health departments, clinics, hospitals, community pop-ups) within their state providing them.

*I know that…yeah, I know a little bit, I guess because I was looking into maybe getting it. I know that they are only, you know, you can only get it at certain places, like not even in every county. But, you know, some counties get together and it’s only at one location. I know that it’s only for those who are very, at least from what I know, very high risk. So, like they ask you a bunch of questions to make sure you are a very high risk. And yeah, that’s what I know… I’m just going to make a comparison here, like to get my… my COVID vaccine, I just, I mean, I just had to take yeah, it’s like a maybe a 20-minute walk from where I am, you know, like it’s in the closest health…health clinic here in my town.***– Hispanic/Latino cisgender gay man**.

However, some men discussed having the resources to overcome and navigate the geographic constraints of limited mpox vaccine accessibility, primarily through their social networks.

*So, I had two appointments, actually. The first… I live in Rhode Island. So, when things started to pick up there, it wasn’t we didn’t have access to a vaccine here. A lot of my social life is in Boston, so in Massachusetts. And they were offering a vaccine there. I called Fenway Health, I think is what it’s called and set up an appointment there. And I just lied about everything. And I gave them, like an address in Boston. I have a lot of friends who live there, so I didn’t really need to make anything up. I’ll make up an address. And then when they asked about the questions related to, like, having multiple sexual partners and stuff, I lied about that too. I was like yeah sure I’m having sex all the time. So that was that. And then but I couldn’t make the appointment on the day that I was supposed to go. So, I did have a trip to New York with some friends and we spent some time in Fire Island. And there they had a clinic going for people who are coming on to the island to get the vaccine.***- Hispanic/Latino queer man**.

While some romantic partners served as a support in getting the vaccine, partners were also a network as a means for accessing certain privileges to streamline the process to receive the vaccine.

*But I know, at least for me, it was very difficult to try to find and secure an appointment to get this monkeypox vaccine. I know at least in the state of Connecticut it was very challenging because you have to fill out a questionnaire online. And I know if you just answered one question that was like outside the parameters, the survey would like automatically cut off and you couldn’t…you couldn’t like sign up for a spot to get the monkeypox vaccine. So, I actually ended up getting the vaccine in New York City. My boyfriend knows somebody that works in the New York State Department that was able to get us the spots to get the monkeypox vaccine. So, I guess I was a little bit surprised of like how difficult it was to get this vaccine, even though, you know, these health care agencies, you know, the CDC and the World Health Organization are pushing so hard to get these vaccines out.***- White and Hispanic/Latino cisgender gay man**.

Structural procedures enforced by federal public health and medical institutions created compounding barriers to accessing the mpox vaccine. This ranged from challenges in making an appointment, inconsistent or lack of transparent eligibility criteria, or increased shortages of the vaccine in their location.

*So, I actually waited a little too long to get my first. We our Department of Public Health ran out of the first dose of vaccines when it came around to me getting my vaccination. I had actually end up having to wait about a week for them to get resupplied. The it was interesting filling out the online survey because they the questionnaire specifically targeted homosexual men and it did feel weird to have to. I mean, this is anonymous. It felt weird to have to lie about, you know, the number of sexual partners I’d had in a week. But in order to get approved, you had to tell them that you were having sex with multiple people within a week and that those people were having sex with multiple people.***- White cisgender man**.

### Intersectional mpox-related stigma on vaccine hesitancy and mistrust

Mpox vaccine hesitancy was informed partially due to men’s lack of perceived risk of acquiring mpox or not recognizing mpox as a burden. Vaccine uptake was not only shaped by men’s sense of mpox risk but also affected by some form of privileged social position, such as occupational hierarchies (e.g., being able to work remotely avoiding in-person contact, having access to medical care if needed) and having social mobility (e.g., network of resources that grant agency) that enabled them to desensitize from being susceptible to mpox or feel unmotivated to get the vaccine.

*I mean, the only reason why I just haven’t gotten it yet is because I just don’t know if it’s really that big yet. Like, it’s kind of like, you know, like even with like COVID like I was hesitant to get it and so it got even bigger. I don’t know where to receive it. I would probably just reach out to my doctor. I mean, and in [city] there is like the [organization] Center, which will give you information, if I could, reach out to them, I’m sure they’ll be able to help me figure it out.***– White cisgender gay man**.

*In terms of like feelings. Not that I didn’t take it seriously, but I just didn’t feel concerned about it. I knew that I was kind of locked up in my house all summer because I was finishing my master’s, and I was taking three courses. So, it was like, I’m not going anywhere. I’m not really seeing anyone. So, you know, it could be it could be out on the streets, right, right across the street from me. And I wouldn’t know. So, I really, really wasn’t a level of concern there.***– White cisgender gay man**.

On the contrary, men also took advantage of their privileged social locations (e.g., being in a relationship with a partner who is supportive of getting the vaccine, access to vaccines) to acquire the vaccine. For instance, receiving the mpox vaccine was a means for feeling safe, while enabling them to continue participating in certain LGBTQ + events and remaining protected at their employment.

*It was my partner’s idea to get it, which did make me kind of like question that. I was just like, uh, okay. But anyway, like I said, I think it’s just because she cares about me and us and you know, I don’t think that she’s cheating or anything like that. I just think that, you know, she just…in case it became like a bigger outbreak and became like a general public issue, maybe that was her reasoning behind it. So anyway, I don’t know exactly how we got signed up for it, but she signed up for it and we went to this place near our house. It’s like one of those public health centers where you get vaccinated.***– White cisgender bisexual man**.

However, some men also explained that despite their confidence in the mpox vaccine, they had speculations about the vaccine based on their mistrust of federal health institutions. Particularly, the men in our study also expressed their concerns in the ways in which these institutions have went about mpox preventative messaging as well as the transparency of the mpox vaccine and its efficacy and risks. Thus, participants have discussed their reliance on people within their social media network and other health-related news for mpox-related information.

*I’m very pro vaccine. I will say I’ve heard a lot. For a while there, I think they were like splitting the dose and only giving one dose*…*I don’t quite trust the people who are in charge in this sort of area. And, and so, yeah, most of my information from the vaccine came from like folks who I follow on Instagram and Twitter who were getting it or the CDC, and um the news. But I really there was not much like, “hey, I got the vaccine.” That’s it. Like, I didn’t hear really anything after that. And I think that’s what that was one of my concerns of like are folks getting side effects or is it actually working? What is the efficacy of it? And that I don’t think was portrayed really at all. There’s just go get it. And I think it’s. Like, is it better to just put yourself in less risky situations? Or is it better to get the vaccine? And I think that every piece of information should be included there. Again, very pro-vaccine. But like they. I feel like it, it just wasn’t communicated well.***– White cisgender gay man**.

Targeted mpox vaccine messaging also fed potential conspiracies about the vaccine and how it made men speculate it as something that may be re-purposed to exercise anti-gay agendas by institutions.

*Like me and my partner kind of jokingly, but also half seriously had a conversation about like what if this vax, you know, like the conspiracy theory behind it, like what if this vaccine is actually meant to target the gay community? And what if it’s, you know, labeling us and or like causing some epigenetic change in our bodies so that we’re not gay, you know, or whatever? I kind of went down the list of conspiracies that you could you could formulate from this.***– White cisgender man**.

Men also shared their sentiments on the anti-gay narratives manifested through mpox preventative messaging. It was clear that health messaging was not only conflating mpox with sexual identity but also highlighted the lack of transparency and consistency in information on preventative practices for mpox that restricted GBSMM from protecting themselves and making health-decisions that empowered them.

*I mean, I think there’s definitely been a lot of polarization in terms of like, I don’t know, like I’ve heard a lot of people talking about like that op-ed that was published about like telling people basically to like stop having sex. I mean, I don’t even know if that’s necessarily what it was saying, but I think that there are like different camps of people that have definitely come out and have been very vocal, one of which is people saying that they’d like gay men to be very careful about like who they’re having sex with, like practice abstinence, like that kind of stuff. And another saying no, like gay men don’t necessarily need to change their behavior, it’s more like this is lack of government oversight leading to this. And obviously there are a lot of people in the middle. So, I mean, I think that just like that debate within the community has definitely been spurred as a result.***– White cisgender gay man**.

Participants also discussed the ways in which the exacerbated stigmatization and homophobic discourse of mpox-related messaging operated across other non-health institutions. In particular, they discuss mpox as a symbolic consequence of religious immorality for being gay or part of the LGBTQ + community. Men also share that enduring religious backlash for being LGBTQ + is not uncommon or a new experience, such as federal politicians as stigmatizers disseminating anti-gay information that puts blame on LGBTQ + people for mpox.

*I kind of take this on more of like, I guess a religious standpoint and like other religious people saying this is like God or Allah, the higher power punishing you for your sins or something like kind of putting the blame on LGBTQ people being like, well, you know, this is what you get for this and that or something. Which, I mean, we’ve all heard it before. We’ve all dealt with it like, okay, you know, you can believe that, but you’re wrong.***– White cisgender gay man**.

*Well, I think it depended on where which media you watched. Yeah, I think I like to see, especially when there’s something like this, like how both sides are covering it, mainly just to feel my anger, I guess be cynical, but like on the right, it was very much and I mean, Marjorie Taylor Greene was talking about how like this is like. I don’t know, something with God coming to all the sin, but like, very much targeted. It’s your fault, LGBTQ people. Like you did this to yourselves, and that’s–I don’t think that’s productive at all…That’s not that’s not smart messaging. And I saw a lot of people I mean, we had Congress people pushing that narrative. And that’s that’s just really dangerous, really, for the next one, because there will be another outbreak of something.***– White cisgender gay man**.

### Call to action and recommendations

Men recognized the nuance of mpox considering the importance of sharing mpox information and reaching communities that are most affected by it, while also avoiding the coating of stigmatizing marketing. Participants felt optimistic that culturally acceptable health marketing would help encourage them to seek health care and become better informed about how to protect themselves from mpox.

*It’s kind of a double-edged sword. I think, like I said, from the very beginning, the LGBT community was being heavily targeted as spreading this disease. But I think one good that might have come of this is hopefully having an LGBT community totally vaccinated against monkeypox. Again, it wasn’t done in the best way by targeting them and kind of alienate…alienating them as people for spreading this disease, but I hope it gave them the opportunity to seek out health care and to ask questions and to be…to be educated members of the public in terms of health care. And I hope it does lead to, you know, a lot of vaccines among that community.***– White and Hispanic/Latino cisgender gay man**.

*I did like that, you know during Pride when it was happening in certain areas, like a lot of places were going to those types of events to try and help people get vaccinated a little bit faster and like think that that’s amazing for the community and for people at large, but the fact that it was still only targeted towards like this one like subset group when like, you know, places are still having like their own, like little town, like festival or whatever. Like, why not do it at those things too?***– Black cisgender queer man**.

Altogether, anti-LGBTQ + discrimination reinforces the erasure of LGBTQ + communities from accurate health campaigning. Participants illuminated the potential benefits that representation of LGBTQ + professionals and knowledge producers within departments of public health could have in order to deliver non-stigmatizing health messages.

*You know, I thought it was pretty good. You know, I think there’s that delicate balance of recognizing that monkey pox is disproportionately affecting LGBTQ plus folks without saying, oh, because they are LGBTQ, plus this is why they have it. So I think that they were given somewhat of a communications nightmare from the jump with it. And, you know, I do think that it was fairly positive, again, and just the fears that that I have seen, especially I know that our like my school luckily we have a department of public health and we also have some LGBTQ plus specific health researchers. So they were also very integral in creating some of that messaging, which was helpful because again, it didn’t necessarily stigmatize. So yeah, I mean, I think it’s pretty positive from what I’ve seen, especially pretty recently.***– Hispanic/Latino cisgender gay man**.

Despite experiencing the institutionally enforced homophobic mpox discourse, men discussed their agency in continued empowerment because of strong cultural ties and community resistance against anti-LGBTQ + conditions.

*I’m not like, I don’t know, like those comments like that and things like that don’t necessarily impact me in that way. Like, and I think that maybe has to do with. Where I’m at and being like the comfort that I have in my like sexuality and stuff like that. So I’m not really taken aback by things. I think I’m just like accustomed and used to it. I know that like if I was younger and hearing folks talk about, you know, monkey pox coming from like the gays and all that, I think I would be impacted a lot more. But I’m at the point in my life where I was just like, okay, cool, that’s fine. I’ll do what I need to do to take care of my my own health.***– Hispanic/Latino cisgender queer man**.

## Discussion

This study was among the first to qualitatively contextualize the experiences of the mpox outbreak among young GBSMM in the US. Informed by intersectionality, the present study assessed the social, political, and structural drivers that shaped the lived experiences and perceptions of mpox among GBSMM. Informed by intersectionality [29–31] to yield attention to the sociostructural power relations that shaped GBSMM’s experience with mpox, we identified four overarching themes on intersectional mpox-related stigma: (1) understanding and conceptualizing mpox, (2) vaccine availability and accessibility, (3) vaccine hesitancy and mistrust, and (4) call to action and recommendations. Consistent with prior studies, there are significant factors that resulted in the dissemination of stigmatizing health messaging and impediments in access to mpox vaccines among GBSMM [6, 52]. Through an intersectional analysis of mpox stigma, the narratives and lived experiences of GBSMM demonstrated how interlocking systems of power (e.g., government, media) and oppression (i.e., heterosexism, homophobia, spatial-economic marginalization) may forge challenges in developing effectively tailored and non-stigmatizing health risk messaging, and mpox vaccine access and uptake. Yet, these stories discern the resilience and resourcefulness of GBSMM in the wake of the mpox epidemic.

The GBSMM in our study elucidate new structural gaps related to mpox and existing hegemonic paradigms, including place-based inequities, health risk communication marketing and institutional mistrust. Even prior to mpox, GBSMM remain disenfranchised communities that have lower utilization of health services [53, 54]. These existing gaps were consistent with the place-based inequities GBSMM in our study experienced in navigating the mpox vaccine. Despite pervasive intersectional mpox stigmatization among GBSMM, some of our participants share nuanced experiences with mpox that were largely shaped by their privileged social locations. For instance, those in our study who received the vaccine mentioned the role of their social networks (e.g., friends and romantic partners) and occupational benefits as enabling factors to get the vaccine. Thus, mpox accelerated existing power asymmetries within GBSMM, who are situated in more privileged classes that supply certain social and economic advantages (e.g., supportive partner, access to health care, flexibility with remote work). In turn, recognizing the neglected conditions (e.g., having a job with higher exposure to mpox, lack of health care access and literacy) other GBSMM may have experienced are important considerations for ongoing and future vaccine-uptake interventions to consider [55, 56]. These determinants are coupled with the disconnect between government mpox messaging that emphasizes the heightened risk of mpox acquisition among GBSMM and the opacity of mpox vaccine eligibility criteria; our participants crystalize the infrastructure gaps that leave GBSMM behind.

The use of health communication marketing efforts has been documented as an important tool for advancing public health emergency guidelines, but deep sociostructural processes such as heterosexism and homophobia remain embedded in these interventions, ultimately reinforcing health inequities among GBSMM. Our discussions with GBSMM underscore the need for capturing and centering their lived experiences but also done so without reinforcing the narrative that they are responsible for the spread of mpox. This salience of mpox stigma and homophobic risk communication efforts emphasizes the need for alternative non-stigmatizing approaches to sexual health equity among GBSMM. This is consistent with previous literature of barriers related to vaccine uptake during public health emergencies, such as the COVID-19 pandemic as these communities continue to resist historical and ongoing medical trauma [57, 58]. This apprehension is also suggested to be rooted in historical labeling practices of GBSMM being more likely to engage in “risky” behaviors or their identities socially attached to certain diseases, like HIV/AIDS.

Given the historical context of stigma and the HIV/AIDS epidemic [59], participants discussed parallels between mpox and stereotyping LGBTQ + communities. These findings remain consistent with large cross-sectional and social media mining studies in which online messaging of mpox was disconnected from non-factual, politically biased and heterosexist [5, 60]. Studies have also reported that the nature of these messages persisted even after the decrease in the incidence of mpox. Thus, concerns for long-term psychosocial stressors that compound on pre-existing embodied inequalities as well as in result prolonging mistrust and underutilization of public health services [24]. Because mpox was predominantly impacting GBSMM communities, some participants outlined the juxtaposition of the benefits that came of the wide dissemination of tailored mpox messaging for the LGBTQ + communities to protect themselves but also recognized the negative consequences that occurred from the nature of mpox messaging. Thus, institutions of health must discontinue the use of deterministic language that conflates disease risk with social identities. This is necessary in developing non-stigmatizing preventative protocols and delivering tailored health risk messaging. Our participants emphasize the significance of how the portrayal of GBSMM in these contexts can worsen existing spatial and sociopolitical inequities that disempower GBSMM from safeguarding themselves during public health emergencies. Additionally, the GBSMM in our study underscore the necessity for increased LGBTQ + empowerment and strengths-based approaches within the media and health institutions to advance more culturally acceptable and non-stigmatizing health messaging. This representation is crucial for challenging stigmatizing representations of GBSMM and dismantling structural homophobia.

While our sample comprised of 33 participants was sufficient for the qualitative nature of this study, its generalizability is limited. However, the nature of qualitative scholarship, such as this enables us to fulfill our objective to unpack the intimate and nuanced lived experiences and stories of mpox among GBSMM. This sample was purposively recruited among participants within an existing study that captures GBSMM within the Northeast and South regions. While the stories of our study sample of GBSMM provides important directions for informing future strategic and ethical health praxis, this sample may not be representative of the larger GBSMM community as majority of the sample was racialized as non-Hispanic White and identified as cisgender gay. Given the temporal implications of the epidemiological curve of mpox, the experiences of GBSMM that were conducted during the height of the outbreak may have varied greatly compared to responses that were held at a later time period where mpox incidence and urgency significantly declined in the US.

## Conclusion

Overall, our sample of participants’ stories illuminate several areas relevant to advancing structural health risk communication and addressing misinformation informed by homophobia. This study emphasizes the priorities for destigmatizing and being critical of our collective approach for prevention during public health crises and efforts to curb health inequities in historically oppressed populations, like the LGBTQ + community. While the burden of abolishing issues, such as mpox-stigma should not be on individuals and scholars of the LGBTQ + community alone, we highlight the importance of centering their voices who are often underrepresented in this work. Therefore, it is crucial that researchers continue to uplift the lived experiences of LGBTQ + communities to advance rapid, yet equitable and just health prevention interventions.

### Electronic supplementary material

Below is the link to the electronic supplementary material.

Supplementary Material 1

## Data Availability

The dataset is qualitative and includes highly sensitive quotes from which our participants are potentially identifiable. Thus, for this reason, the raw dataset will not be available for distribution. Additional quotes supporting each theme will be made available upon request from trace.kershaw@yale.edu.

## Abbreviations

Mpox: Monkeypox
GBSMM: Gay, bisexual, other sexual minoritized men
#MVMNT: Men’s Voices on Mapping, Neighborhoods, and Technology
