# Supplementary material for: “It’s another gay disease”: an intersectional qualitative approach contextualizing the lived experiences of young gay, bisexual, and other sexual minoritized men in the United States during the mpox outbreak
Source: BMC Public Health. 2024 Jun 11;24:1574. doi: 10.1186/s12889-024-19062-z (PMC11167942; doi:10.1186/s12889-024-19062-z)
Supplement: Supplementary file 1 — Supplementary Material 1 [file 12889_2024_19062_MOESM1_ESM.docx]

Script

***In the following section, we are interested to learn what you know about the recent Monkeypox outbreak in the United States. We will ask you about general questions about the outbreak, your information sources, personal susceptibility, opinions on monkeypox vaccines, and your overall perception about the impact of this event in society.***

1. What are your current thoughts about the Monkeypox outbreak?
2. What have you heard about monkeypox?
3. What do you think about these messages?
4. How has what you have heard about the outbreak made you feel?
5. How susceptible do you think you are to Monkeypox (how likely is it that you will be exposed)?
6. What consequences do you foresee if you were to get Monkeypox?

1. What have you observed about how people with Monkeypox have been viewed or treated?
2. If you have known anyone who has gotten Monkeypox, how have they been treated or viewed by others?
3. How do you think other people would see you or treat you if you were to get Monkeypox?
4. What do you know about the monkeypox vaccine?
5. What would help you decide whether or not to get the vaccination?
6. Where would/did you go if you got the vaccine?
7. What changes are you considering or making to protect yourself from Monkeypox?
8. What things have motivated you or might motivate you to make changes or protect yourself?
9. What kinds of communication are you having with your sexual partners about monkeypox?
10. There have been discussions in the media about how monkeypox is specifically affecting the LGBTQ community. What effect do you think this will have on the community?
